# Genetic Diversity and Structure of the Main Danubian Horse Paternal Genealogical Lineages Based on Microsatellite Genotyping

**DOI:** 10.3390/vetsci9070333

**Published:** 2022-07-01

**Authors:** Georgi Yordanov, Ivan Mehandjyiski, Nadezhda Palova, Nedyalka Atsenova, Boyko Neov, Georgi Radoslavov, Peter Hristov

**Affiliations:** 1 Bulgarian Horse Breeding Society, 1756 Sofia, Bulgaria; nak.sofia.ofis@gmail.com; 2 Research Center of Stockbreeding and Agriculture, Agricultural Academy, 4700 Smolyan, Bulgaria; bg_porodi07@abv.bg; 3 Scientific Center of Agriculture, Agricultural Academy, 8300 Sredets, Bulgaria; nadejda_palova@abv.bg; 4 Department of Animal Diversity and Resources, Institute of Biodiversity and Ecosystem Research, Bulgarian Academy of Sciences, 1113 Sofia, Bulgaria; atsenovaned@gmail.com (N.A.); boiko.neov@gmail.com (B.N.); g.radoslavov@gmail.com (G.R.)

**Keywords:** genetic diversity, microsatellite DNA, population structure, horse sire lineages

## Abstract

**Simple Summary:**

The Danubian horse was created on the former Klementina stud farm near Pleven to satisfy the demands of the Bulgarian Army for light draft horses and to improve the working capacity of the local horse population. The privatization of the Klementina stud farm in the late 1990s and the lack of economic activity have led to a sharp reduction in the number of mares and stallions and their sale to private owners. At present, only six of the main paternal lines which participated in the creation of the Danubian horse breed are preserved: Zdravko, NONIUS XVII-30, Torpedo, Lider, Kalifa, and Hrabar. This is the first study on the genetic and population diversity of the Danubian horse paternal lines based on microsatellite markers (STRs). The results showed that the studied Danubian horse population was characterized by a high level of genetic diversity with a medium value of 0.84. The fixation index (*F*_ST_) was 0.08 for all studied markers, which is indicative of the low genetic differentiation of the Danubian horse population. Our analysis also confirmed the low level of inbreeding and heterozygous deficiency among the animals selected from the six paternal lineages of the Danubian horse. The present research could be helpful for the development of breeding and conservation programs for the Danubian horse, as well as for making informed decisions on the management of paternal lines.

**Abstract:**

The Danubian horse, together with the Pleven and the Eastern Bulgarian horse breeds, is one of the modern breeds in Bulgaria. The objective of this study was to compare the genetic structure and genetic diversity of six paternal genealogical lineages of the Danubian horse breed (Zdravko, NONIUS XVII-30, Torpedo, Lider, Kalifa, and Hrabar). In total, 166 individuals from the six genealogical lines were investigated, based on 15 STR markers (short tandem repeats, also known as microsatellites). In total, 184 alleles were found in the six populations, using 15 microsatellite loci. The mean number of alleles, the effective number of alleles, and the polymorphic information content (PIC) values per locus were 12.28, 9.48, and 0.73, respectively. In a comparison of the allelic diversity among sire lineages, the highest genetic diversity (Na) was observed in Lider and Kalifa (14.60 ± 0.21), while the lowest value of this parameter was observed in the Zdravko lineage 4.20 ± 0.35. The largest genetic diversity was found in loci HMS3 and HMS7, with 13 alleles, and the smallest polymorphism was noted for the locus ASB17, with 10 alleles. The level of observed heterozygosity was in the range of 0.65 ± 0.069 for the Zdravko lineage to 0.93 ± 0.01 for the Torpedo lineage. The expected heterozygosity level range was from 0.57 ± 0.048 to 0.91 ± 0.01 for all horse lineages. Structure analysis revealed three main gene pools in the study population. The first pool included the Zdravko lineage; the second had the NONIUS XVII-30, Torpedo, Lider, and Kalifa lineages; and the third defined the Hrabar lineage, which was significantly differentiated from the other genealogical lineages.

## 1. Introduction

Considering the ever-changing dynamics of today’s reality, it is hard to anticipate what kind of animal breeds will be necessary tomorrow, what their productivity will be, what human needs for food or other demands will be, and what will be required by society and the common people thereof [1,2,3]. For many years, the creation of different horse breeds depending on human needs has made it possible to use the horse in many areas of our lives such as war, transport, religion, trade, agriculture, communication, sports, and recreation [4,5,6]. Nowadays, many of these areas of horse use have ceased to exist, and horses are currently bred mainly for sports and sports tourism. This change in horse use is due to several reasons: first, riding populations as well as racing breeds are increasing worldwide, while many autochthonous breeds are currently threatened with extinction [7,8]; secondly, horse breeders are radically changing their breeding strategy to meet these needs [9,10]. In addition, there is the frequent practice of the so-called out-crossing process, i.e., combining different breeds in the breeding process in order to increase genetic diversity and improve productivity among local and introduced horse breeds [11,12].

**Figure 1:**
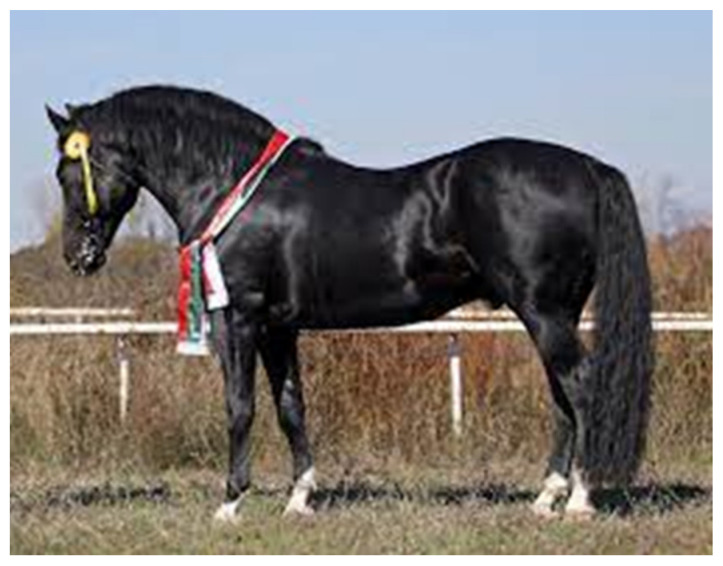
A typical representative of the Danubian horse breed (photo by Georgi Yordanov).

One of the modern horse breeds in Bulgaria is the Danubian horse (Figure 1).

**Figure 2:**
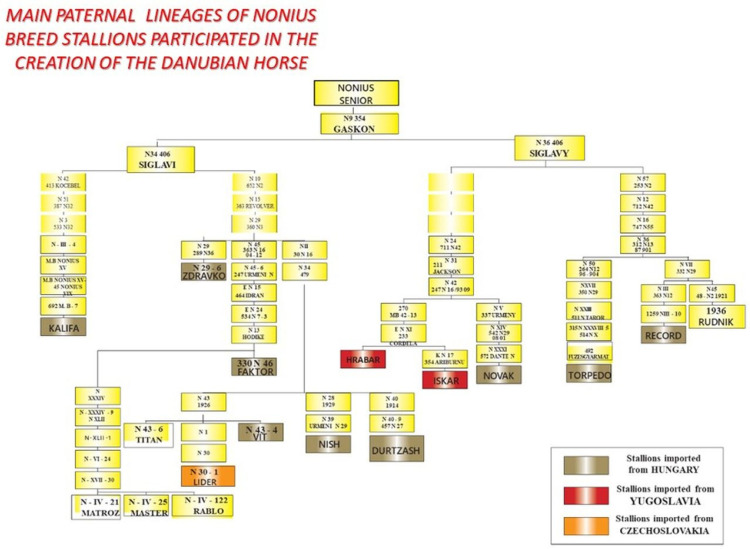
Genealogical lineages of the Nonius founder stallions participating in the creation of the Danubian horse in Bulgaria.

This horse was officially recognized as a breed in 1951 [13]. The Danubian horse was created in the Klementina stud farm in the village of Pobeda (Pleven region). The basis of the tribal nucleus was stallions and mares of the breed Nonius, imported from Hungary (from the Mezohegyes stud farm) and also from the former Republic of Yugoslavia and the Czechoslovak Republic in 1956 (Figure 2) [13]. From the beginning, purebred breeding of the imported stallions and mares from the Nonius breed was applied [14]. Simultaneously, there was also crossbreeding with Nonius stallions and partial reproductive crossbreeding, known as “grading-up”, i.e. sires of the Nonius breed were continually backcrossed with females of the previous generation of local and improved mares [14]. These three breeding methods, along with strict selection, have contributed to the creation of a specific kind of Bulgarian Nonius, which differs from the Nonius breeds in Hungary, Romania, and the former Republic of Yugoslavia [15]. According to data published by the National Association of Horse Breeding, in 2019, the number of horses controlled by the Association which were registered in the studbook and the register of the breed was 250 in total. From all the founder Nonius sire lineages shown in Figure 2, only six paternal lineages of the Danubian horse are available today: Zdravko, NONIUS XVII-30, Torpedo, Lider, Kalifa, and Hrabar.

The population of the Danubian horse in Bulgaria is on the verge of the critical minimum (~350 individuals), which threatens its existence. This requires urgent plans in order to prepare a selection program for an adequate approach to reproduction and the avoidance of inbreeding processes. This determined the purpose of the present study, namely genotyping of representatives of the Danubian horse from six paternal genealogical lines (Zdravko, NONIUS XVII-30, Torpedo, Lider, Kalifa, and Hrabar), based on 15 microsatellite markers, for clarification of the genetic structure and biodiversity of the breed.

## 2. Materials and Methods

### 2.1. Animal Welfare and Ethical Statement

All experimental procedures were reviewed and approved by the Animal Research Ethics Committee of the Bulgarian Food Safety Agency (BFSA) (Art. 154 of the Law on Veterinary Activity), in accordance with European Union Directive 86/609.

### 2.2. Sample Collection

Hair samples from the mane and/or tail (containing about 50 hair follicles from each animal) were collected from 166 animals from Danubian horse representatives of six paternal genealogical lineages (Zdravko, n = 11; NONIUS XVII-30, n = 21; Torpedo, n = 29; Lider, n = 33; Kalifa, n = 34, and Hrabar, n = 38). The samples were collected in a way to ensure that each extracted hair contained a follicle and a sufficient length for the shaft samples. Most of the samples were from private tribal horse farms in the Bulgaria stud and the Klementina stud farm in the village of Pobeda (Pleven region, Bulgaria).

For all Danubian horse individuals analyzed in this study, to trace the genealogical structure, the genealogical data were obtained from records in studbooks located in the Executive Agency for Selection and Reproduction in Animal Husbandry, the pedigree lists of the Klementina stud farm, the breeding program for the Danubian horse breed, and the database of the National Horse Breeding Association. We eliminated all samples with a doubtful or excluded pedigree. The information gathered showed that currently, the largest number of licensed and certified stallions for breeding is for the Torpedo line—with 6 stallions, followed by the lines of Kalif, Hrabar, and Lider, with 5 stallions each. Regarding the Zdravko line, there is only a single stallion left: Zahir. In the genetic bank of the Executive Agency for Selection and Reproduction in Animal Husbandry, there is cryopreserved semen from the stallion Zaher (from the Zdravko lineage), which will be used for the development of the lineages.

### 2.3. DNA Extraction

Total DNA was extracted from hair follicle and hair shaft samples, using a GeneMATRIX Tissue DNA purification kit (Cat. No. E3550, EURx Ltd., Gdansk, Poland), according to the manufacturer’s instruction. Briefly, 1–3 hair follicles or 3 pieces of hair or, in cases when no hair follicles were available, shafts of approximately 0.5 cm in length were cut and placed in an Eppendorf tube. After that, they were mixed with 350 μL of the buffer Lyse T, 20 μL of 1 M DTT, and 20 μL of proteinase K and incubated overnight at 56 °C with shaking. The quality and quantity of the isolated DNA were checked by 1% agarose gel electrophoresis and then visualized om UV transilluminator gel documentation systems after staining with SimpliSafe™ (Cat. No. E4600; EURx Ltd., Gdansk, Poland). The isolated DNA was stored at −20 °C before analysis.

### 2.4. Microsatellite Markers

We analyzed a total of 15 ISAG [16] recommended markers: 12 markers from the ISAG core panel (AHT4, AHT5, ASB2, ASB17, ASB23, HMS2, HMS3, HMS6, HMS7, HTG4, HTG10, and VHL20) and another three from ISAG’s extra panel (HMS1, HTG6, and HTG7) (Table 1).

### 2.5. PCR Amplification and Fragment Analysis

All samples were sent to the GeneControl GmbH laboratory (Grub, Germany), where PCR amplification and fragment analysis were performed.

### 2.6. Statistical Analysis

GenAlEx 6.5 (New Brunswick, NJ, USA) [24] was used to calculated the mean allelic patterns across populations and the Hardy–Weinberg equilibrium (HWE). A principal coordinate analysis (PCoA) to reveal the major patterns of genetic variability and clustering of breeds, based on an *F*_ST_ matrix, was also computed with GenAlEx 6.5. The polymorphic information content (PIC) of each locus was calculated using PICcalc [25]. The fixation indices (*F*_IT_, *F*_IS_, and *F*_ST_) were obtained by Wright’s F-statistics [26] using POPGENE software [27]. Nei’s genetic diversity (H_T_), the diversity between populations (*D*_ST_), and the coefficient of gene differentiation (*G*_ST_) values were calculated with FSTAT 2.9.4 [28].

Population structure was evaluated using STRUCTURE 2.3.4 software [29]. The number of presumptived clusters (K) was run from 2 to 12, where K was the number of tested clusters. Ten iterations were performed for each K value. All runs were performed with a length of 50,000, followed by 150,000 Markov chain Monte Carlo (MCMC) repeats after burn-in, with 20 replicate runs for each K, using an admixture model and independent allele frequencies. The results were processed by STRUCTURE HARVESTER [30] to determine the optimal number of groups (K), the log-likelihood coefficient (Delta K) [29], and the ΔK value of Evanno et al. [31]. The software package Clumpak (http://clumpak.tau.ac.il/, accessed on 13 June 2022) was was used to identify most probable values of K [32]. The DISTRUCT application was used in order to display the results graphically [33].

## 3. Results

### 3.1. Polymorphism of Microsatellite Markers

In total, 184 alleles were identified in the studied 166 animals within six paternal genealogical genotypes at 15 microsatellite loci. All markers were found to be polymorphic in the population of the Danubian horse breed (Table 2). The mean number of alleles ranged from 11.2 at locus AHT4 to 13.3 at loci HMS3 and HMS7, where the mean number of alleles (Na) was 12.29 and the effective number of alleles (Ne) was 9.48. The expected heterozygosity (He), which is acknowledged as the best parameter of genetic diversity in a population, varied from 0.75 in locus AHT4 to 0.89 in locus HTG10, with an average He of 0.84 across the genealogical lineages for the analyzed 15 microsatellite loci. The observed heterozygosity (Ho) fluctuated from 0.75 in locus AHT4 to 0.96 in locus ASB23, with a population mean of 0.66 (Zdravko lineage) and 0.94 (Torpedo lineage), indicating that all studied lineages are characterized by considerable genetic variability. The polymorphic information content (PIC) varied from 0.65 for the marker HMS1 to 0.81 for the AHT4 locus. The average PIC for the 15 microsatellite markers was 0.73, and there were no markers with a PIC of less than 0.5; hence, all loci were found to be highly polymorphic. Shannon’s information (diversity) index (I), which is an indicator of the genetic diversity of a population, ranged from 1.85 in locus ASB17 to 2.42 in the HMS7 marker (Table 2). The average value of I for all six genealogical lineages was 2.22, which means that increasing entropy emphasizes the most abundant alleles.

The average *F*_IS_ value was −0.043 (*p* = 0.002) (Table 2). There were no markers which revealed *F*_IS_ values higher than 0.1. The *F*_IT_ fixation index, used for measuring the heterozygosity loss of individuals with respect to the overall population, was 0.037 (*p*  =  0.004), showing that there was no excess of or deficiency in heterozygotes over the total population (*F*_IT_ was nearly zero). The mean *F*_ST_ index, which is used for measuring the degree of genetic differentiation among the six lineages, was 0.078, thus indicating that genetic diversity was significantly higher within the genealogical lineages than among them.

The calculated mean *D*_ST_ value, which describes the diversity among the six paternal lineages, was 0.46. The general mean of the *G*_ST_ value determining the genetic differentiation was 0.061, indicating that 6.1% of the genetic variation existed among the lineages and 93.9% of the genetic variation existed within them. Nei’s average gene diversity (H_T_) was in the range of 0.82 and 0.93 (Table 2). This value indicated a high level of heterozygosity in the studied lineages.

Hardy–Weinberg equilibrium tests (HWS tests) were performed for all genealogical paternal lineages, based on 15 STRs markers (Table 3). The obtained results showed that in the Torpedo line only, there was no deviation from the HWS. Significant deviations were observed in the HMS1 locus in the NONIUS XVII-30 (*p*  <  0.05) and Kalifa (*p*  <  0.01) lineages. Deviations were also observed in the ASB17 marker in the Zdravko (*p*  <  0.05) and Hrabar (*p*  <  0.001) lineages. The presence of a heterozygous deficit was observed in the loci HTG6 (Kalifa, *p*  <  0.05), ASB23 (Hrabar, *p*  <  0.01), and HTG10 (Lider, *p*  <  0.05).

### 3.2. Genetic Diversity within and among the Genealogical Lineages

Table 4 shows the main parameters used to assess the genetic diversity of the Danubian horse breed. The mean number of observed alleles varied from 4.20 in the Zdravko lineage to 14.60 in the Lider and Kalifa lineages. It is interesting to note that we observed one private allele only in the Kalifa lineage, with a frequency of 2.9% (ASB17 locus) in all 15 studied loci (Table 4). The Ho fluctuated from 0.65 in the Zdravko lineage to 0.94 in the Torpedo lineage, while the He indicated the lowest value again in the Zdravko lineage (0.57) and the highest rate (0.91) in the Lider and Kalifa lineages. It is noteworthy that only in the Zdravko and Kalifa lineages, the values of Ho were lower than those of He (Table 4). This indicates that heterozygous deficiency is present in this lineage, which is most pronounced at the AHT4 locus. The opposite trend (He > Ho) was observed for all other lineages.

The number of different alleles with a frequency of ≥5% showed the lowest value in the Zdravko lineage (only three alleles), while in the Torpedo lineages, the number was the largest (almost 11 alleles).

We further compared Nei’s genetic distances and the *F*_ST_ values of the studied paternal lineages as a measure of their genetic differentiation (Table 5). From the Nei minimum genetic distance (D_A_) values, it was observed that two pairs of genealogical paternal lineages Lider and Kalifa, and Lider and Torpedo were genetically the closest among the studied lineages (0.171 and 0.188, respectively). The highest genetic distance was determined between Zdravko and NONIUS XVII-30 lineages (1.142).

We also compared the *F*_ST_ values between the studied lineages as a measure of their genetic differentiation (Table 5). The *F*_ST_ values showed a high differentiation coefficient between Zdravko and all other lineages (ranging from 0.103 to 0.122), possibly due to the different genetic structure of the Zdravko lineage. The other genealogical lineages showed low genetic differentiation, with *F*_ST_ values between 0.008 and 0.0354, suggesting a homogenous structure within these lineages.

### 3.3. Genetic Structure and Principal Coordinate Analysis

**Figure 3:**
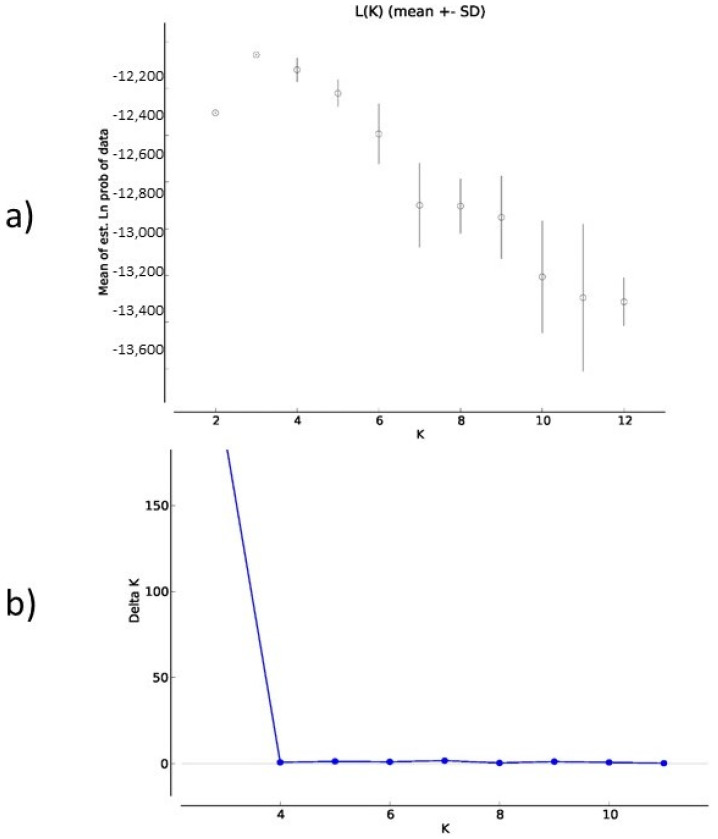
Delta K values of the STRUCTURE analysis of six Danubian horse sire lineages. (**a**) Delta K, calculated according to Evanno et al. [31], is plotted against the number of modeled gene pools (K). (**b**) The highest likelihood and Delta K were observed for K = 3.

**Figure 4:**
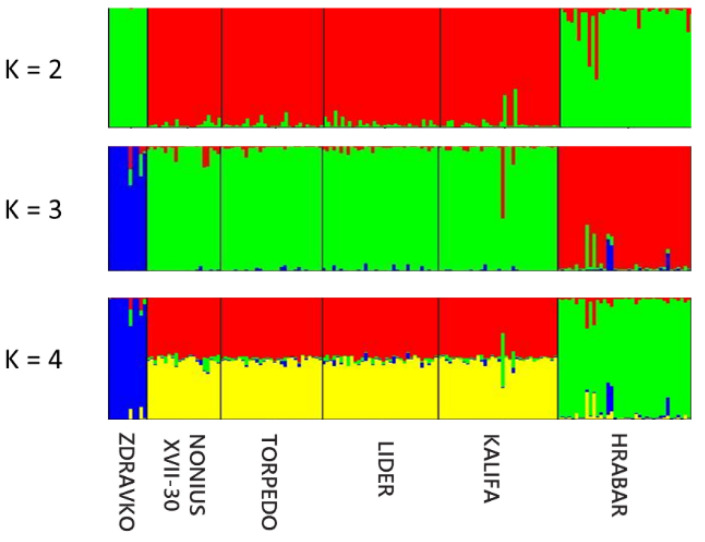
Genetic structure of six Danubian horse genealogical paternal lineages based on 15 SSR marker data. The clustering assignment depended on the Bayesian method under an admixture model obtained with STRUCTURE software. Each individual is represented by a single column that is divided into segments whose size and color correspond to the relative proportion of the animal genome corresponding to a particular cluster.

The genetic population structure of each genealogical paternal lineage was determined on the basis of the admixture level for each individual, using a correlated allele frequency model implemented in STRUCTURE software v0.6.94 (Irvine, CA, USA). The results of Delta K indicated that the optimal number of genetic clusters representing the most similar ancestral breeds was at K = 3 (Figure 3a,b). Figure 3 clearly demonstrates that the studied horse lineages were differentiated by three genetic clusters. The three clusters were made up of the Zdravko lineage in the first; the NONIUS XVII-30, Torpedo, Lider, and Kalifa lineages in the second; and Hrabar in the third cluster (Figure 4). As can be seen from Figure 4, each individual is represented by a single vertical line. The mixed colors and proportional lengths represent the admixture level for the subpopulations of K between 2, 3, and 6. The first genetic pool has individuals mainly of the Zdravko lineage, but also some individuals of all other lineages, with different assignment probabilities (Figure 4). Similarly, many individuals of this gene pool are defined mainly in blue. Very few individuals of the Zdravko lineage had high assignment probabilities to the second cluster (NONIUS XVII-30, Torpedo, Lider, and Kalifa lineages; green color). Alternatively, most individuals of the second pool (green color; K = 3) were solely assigned to the NONIUS XVII-30, Torpedo, Lider, and Kalifa lineages. This gene pool also showed the presence of some individuals which could be assigned from the third cluster (red color, Hrabar lineage). The third genetic pool had Hrabar lineage, with very few individuals with a limited admixture proportion of the second gene pool. The shared proportion of the second gene pool was observed in the other two pools, possibly indicating a common ancestry origin.

**Figure 5:**
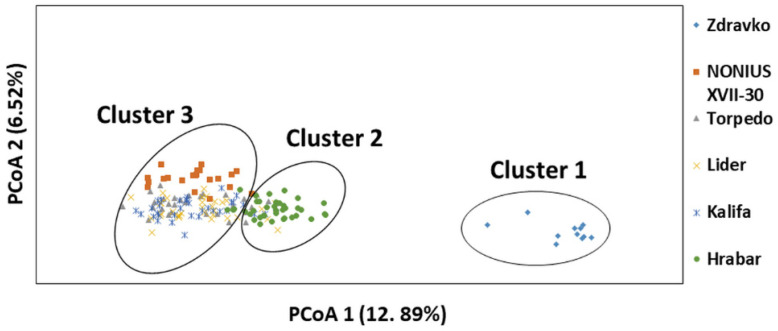
Population structure of six Danubian horse sire lineages genotyped with 15 microsatellites by means of principal coordinate analysis (PCoA), using GenAlEx 6.5 software. Principal Coordinates 1 and 2 were plotted for individuals (the shapes indicate which lineages they were from).

The clustering observed by means of STRUCTURE software was supported by a principal coordinate analysis (PCoA) (Figure 5). The PCoA divided the 166 individuals into three clusters. Principal coordinates (PC) 1 and 2 explained 12.9% and 6.5% of the variance in the genotype data, respectively. The dendrogram divided all Danubian sire lineages into three major clades. Overall, the dendrogram corroborated the STRUCTURE results, where a few individuals assigned to Cluster 3 were also present in Clade 2.

## 4. Discussion

The main aim of the breeding policy of the Danubian horse was to create a horse with a good exterior and constitution to improve the speed traits and movement of horses [34,35]. The main purpose of the breed was focused on four directions: transport, harness, equestrian sport, and riding. It is interesting to note that using stallions outside the Danubian breed for breeding in the population is not considered a mistake, as long as it corresponds with the genotyping guidelines stated in the breeding program.

### 4.1. Population Genetic Diversity of Paternal Lineages in the Danubian Horse

The results of the analysis of the population of the Danubian horse in Bulgaria showed a high level of polymorphism in all 15 studied microsatellite loci (Table 2). In total, 184 alleles were identified, where the average number of alleles per locus was 12. The observed Na is an important component of genetic diversity and is essential in assessing the genetic variability in a given locus. According to FAO recommendations, microsatellite markers represented by more than four alleles need to be used to study genetic diversity [16]. Among the 15 markers used in the study, all loci showed a significantly higher Na, which gives us reason to believe that the microsatellite markers used were suitable for the analysis of the genetic diversity in the population. The overall mean number of alleles was higher than that in the Nonius (7.76 alleles/locus) [36], the Trotter horse populations in Bulgaria (6.46 alleles/locus) [37], and the Bulgarian Thoroughbred horse (5.7 alleles/locus) [38]. The polymorphic information content (PIC) is an indicative parameter of the informativeness of microsatellite markers and their usefulness in diversity analyses of a breed. In our study, the high mean value of PIC (0.73, >0.5) and the high average number of alleles per locus indicated that the panel of 15 microsatellite markers used was suitable for studying the genetic diversity in the Danubian horse breed (Table 2). The mean value of PIC was higher than those observed in the Chinese Guanzhong horse (0.51) [39], the Italian Thoroughbred horse (0.62) [40], and the Polish Konik horse (0.67) The mean value of PIC was higher than that observed in the Chinese Guanzhong horse (0.51) [41] but lower than that in the Turkmen horse population (0.77) [42]. Shannon’s information (diversity) index (I) [43], which is an indicator of the genetic diversity of a population, ranged from 1.85 in locus ASB17 to 2.38 in the INRA005 marker and 2.42 in locus HMS7 (Table 2). The average value of I for the six Danubian paternal lineages was 2.22, which means that increasing entropy emphasizes the most abundant alleles.

The values of the observed heterozygosity (Ho) ranged from 0.75 at the AHT4 locus to 0.96 at the ASB23 marker (Table 2). The expected heterozygosity (He) and the genetic diversity, respectively, varied from 0.74 in locus AHT4 to 0.89 in loci HMS7 and HTG10. It is noteworthy that in almost all the studied loci (13 out of 15), the values of Ho were higher than those of He. This indicates that no heterozygous deficiency was observed at these loci. Heterozygous deficiency (He > Ho) was present at loci HMS7 and HTG6, and was most pronounced at the HTG6 marker. Although some microsatellite markers indicated the presence of a certain level of heterozygous deficiency, in general in the studied population of Danubian horses, there was a high level of genetic diversity with a medium value of 0.84.

The obtained values of the *F*_ST_ index and Nei’s genetic distance (Table 5), which are frequently used as indicators of relatedness, confirmed significant differences among the lineages. According to the generally accepted criteria, the average value of *F*_ST_ at a level of around 0.05 can be regarded as low, and the population of the Danubian horse showed low genetically differentiation for all the studied markers [36]. The *F*_IT_ values were close to zero (0.037) in all microsatellite loci, thus indicating that inbreeding depression is not a threat in the Danubian horse population. The inbreeding coefficient (*F*_IS_) is an indicator of inbreeding among individuals in a population and is considered to be the main reason for deviation from Hardy–Weinberg equilibrium. The values of the *F*_IS_ coefficient vary from −1 to +1, and positive values are an indicator of heterozygous deficiency in a given locus in the whole population [44]. In our study, the *F*_IS_ value was negative for most loci (mean value −0.043), thus confirming the low level of inbreeding and heterozygous deficiency among the animals selected from the six paternal lineages of the Danubian horse (Table 2). The *F*_IS_ index was lower than those observed in the Nonius horse population [36], the Trotter horse population in Bulgaria [37], the Polish Konik [41], and the Czech Haflinger horse [44], etc.

### 4.2. Genetic Differentiation within and between Paternal Lineages in the Danubian Horse

All six studied lineages exhibited a high mean number of alleles (27.6) (Table 4). The smallest number of alleles/locus was observed in Zdravko (4.2), and the largest in the Lider and Kalifa lineages (14.6). In contrast, in Thoroughbred and Trotter horses in Bulgaria, the Na was 5.7 and 6.8 alleles/locus, respectively [37,38]; in the Nonius horse, it was 4.05 alleles/locus [36]; and in the Polish Konik horse, it was 6.16 alleles/locus [41].

The Ho showed the highest value in Torpedo (0.94) and the lowest value in Zdravko (0.65). In contrast, the Lider and Kalifa lineages had the highest He (0.91), indicating that they were the most genetically variable lineages. This was supported by the high value of the I index in the Lider (2.54) and Kalifa (2.53) lineages. The Ho and the He coefficients were also high (above 0.5), indicating a good level of variability across all lineages.

Structure and admixture analyses were used in earlier studies involving different horse populations to provide an appropriate approach to determine ancestral, pure, and hybrid populations [45,46,47]. According to pedigree information, the Danubian horse breed has its own structure, consisting of several main sire lines: the stallions Zdravko and Hrabar; several newer ones (Kalifa, Torpedo, and Lider); and the latest (NONIUS XVII-30), through sons of the stallion Nonius XVII-30 (Matroz, Mester, and Rablo) imported from Hungary in 2014 and 2016 [48].

The results of the STRUCTURE analysis showed that the six genealogical lineages could be clustered into three gene pools (Figure 4). All individuals of the Zdravko lineage were assigned to a separate gene pool, with very few individuals showing a small fraction of admixture deriving from the second cluster. The stallion Zdravko represented a typical Nonius horse imported in 1922 from the Mezohegyes stud farm in Hungary [49]. In general, the breeding activity of his sons was relatively low, and the total number of offspring left by Zdravko was too small. The line is currently threatened with extinction. We consider that the Zdravko lineage formed a separate cluster due to the greatly reduced population size of the lineage, and this type of bottleneck effect may have caused the different genetic structure. The second gene pool (cluster) is composed of four lineages: NONIUS XVII-30, Torpedo, Lider, and Kalifa. The stallion Lider was imported from the former Czechoslovakia in 1956 and was the only purebred Nonius imported into our country, and was related to the most typical representatives of the heavier type of Nonius [15]. All these four lines are typical representatives of the Vernier breed, which most likely makes it possible to distinguish them into a separate cluster. The results also showed that at the individual level the NONIUS XVII-30, Torpedo, Lider, and Kalifa lineages had a few genetic admixtures from the Hrabar lineage (Figure 4). The stallion Hrabar was used in a period when the mares were not sufficiently aligned by type and by origin. A great number of stallions of Hrabar lineage took part in the breeding process in the main areas where the breed is distributed [15]. Thus, it can be assumed that the Hrabar lineage had an influence on the second gene pool, most likely due to the use of the similar genetic profile of the maternal lines participating in the breeding process in the creation of the Danubian horse. The third cluster consists of representatives of the Hrabar lineage. This cluster is clearly differentiated from the other two. In fact, the founder of this lineage, the stallion Hrabar, imported from the former Yugoslavia, was the only representative of the Danubian horse sires that was not a purebred Nonius, since his mother was a local Serbian mare (Navika) [15]. We assume that this is the reason for the grouping of all individuals from the Hrabar lineage in a separate cluster. The obtained data would be useful for a further assessment of genetic variation in Danubian horse breeding in Bulgaria. On the other hand, a few individuals of the Hrabar gene pool show a proportion of admixture from the second gene pool (NONIUS XVII-30, Torpedo, Lider, and Kalifa lineages). This result was probably, first of all, due to shared ancestry (mainly maternal lines) and, secondly, due to the gene flow between the sire lineages being reared in the same geographic areas (the Klementina stud farm).

The results from the PCoA plot analysis support the data from the STRUCTURE analysis (Figure 5). Figure 5 clearly demonstrates the formation of three separate clusters in all six Danubian horse paternal lineages. Cluster 2 (Hrabar lineage) and Cluster 3 (NONIUS XVII-30, Torpedo, Lider, and Kalifa lineages) show the presence of some individuals from Cluster 2 in Cluster 3 and vice versa.

## 5. Conclusions

This study evaluated the genetic diversity and population structure of Danubian horse individuals selected by the main paternal lines. The low *F*_ST_ value of 0.08 in the overall population of the Danubian horse paternal lines suggests that this local breed is not differentiated enough. The specific grouping of the main paternal lineages into three separate cluster suggests the different origin of the Zdravko and Hrabar lineages, and the common ancestry of the NONIUS XVII-30, Torpedo, Lider, and Kalifa lineages. Due to the small number of typical representatives of the Danubian horse, the genetic structure of the breed must be monitored, as certain paternal lines now have an uneven representation. However, if we objectively consider the question of the number of representatives in each male line by stallions, on the one hand, and through mares, on the other, in severely reduced population numbers, there is a negative outlook for the future development of the breed.

## Figures and Tables

**Table 1:** Horse STR information used in the study: Locus name, chromosomal location, repeat structure and repeat sequence, reference, primer sequences, and the size ranges of the amplicons.

Locus	Chrom. Location	Motif	Reference	Primer Sequences 5′–3′	Annealing T (°C)	Amplicon Length (bp)
AHT4	24q14	(AC)nAT(AC)n	Binns et al. [17]	F: AACCGCCTGAGCAAGGAAGT R: CCCAGAGAGTTTACCCT	60	144–164
AHT5	8	(GT)n	Binns et al. [17]	F: ACGGACACATCCCTGCCTGC R: GCAGGCTAAGGAGGCTCAGC	60	126–144
ASB2	15q21.3-q23	(GT)n	Bowling et al. [18]	F: CCACTAAGTGTCGTTTCAGAAGG R: CACAACTGAGTTCTCTGATAGG	55	216–250
ASB17	2p14-p15	(AC)n	Bowling et al. [18]	F: ACCATTCAGGATCTCCACCG R: GAGGGCGGTACCTTTGTACC	60	87–129
ASB23	3q22	(TG)n	Irvin et al. [19]	F: GCAAGGATGAAGAGGGCAGC R: CTGGTGGGTTAGATGAGAAGTC	58	
HMS1	15	(TG)n	Guerin et al. [20]	F: CATCACTCTTCATGTCTGCTTGG R: TTGACATAAATGCTTATCCTATGGC	58	170–186
HMS2	10	(CA)n(TC)2	Guerin et al. [20]	F: CTTGCAGTCGAATGTGTATTAAATG R: ACGGTGGCAACTGCCAAGGAAG	58	222–248
HMS3	9	(TG)2(CA)2TC(CA)n and (TG)2(CA)2TC(CA)nGA(CA)5	Guerin et al. [20]	F: CCATCCTCACTTTTTCACTTTGTT R: CCAACTCTTTGTCACATAACAAGA	60	148–170
HMS6	4	(GT)n	Guerin et al. [20]	F: GAAGCTGCCAGTATTCAACCATTG R: CTCCATCTTGTGAAGTGTAACTCA	60	151–169
HMS7	1q25	(AC)2(CA)n	Guerin et al. [20]	F: TGTTGTTGAAACATACCTTGACTGT R: CAGGAAACTCATGTTGATACCATC	60	165–185
HTG4	9	(TG)nAT(AG)5AAG (GA)5 ACAG(AGGG)3	Ellegren et al. [21]	F: CTATCTCAGTCTTGATTGCAGGAC R: CTCCCTCCCTCCCTCTGTTCTC	55	127–139
HTG6	15q26-q27	(TG)n	Ellegren et al. [21]	F: GTTCACTGAATGTCAAATTCTGCT R: CCTGCTTGGAGGCTGTGATAAGAT	58	84–102
HTG7	4	(GT)n	Marklund et al. [22]	F: CCTGAAGCAGAACATCCCTCCTTG R: ATAAAGTGTCTGGGCAGAGCTGCT	58	118–128
HTG10	21	(TG)n and TATC(TG)n	Marklund et al. [22]	F: TTTTTATTCTGATCTGTCACATTT R: CAATTCCCGCCCCACCCCCGGCA	55	95–115
VHL20	30	(TG)n	Van Haeringen et al. [23]	F: CAAGTCCTCTTACTTGAAGACTAG R: AACTCAGGGAGAATCTTCCTCAG	60	87–105

**Table 2:** Number of identified alleles per locus (Na), number of effective alleles (Ne), polymorphism information content (PIC), observed (Ho) and expected (He) heterozygosity, Shannon’s information index (I), *F*_IT_ (inbreeding coefficient), *F*_IS_ (inbreeding coefficient of an individual relative), *F*_ST_ (fixation index), *D*_ST_ (gene diversity between populations), H_T_ (total expected heterozygosity), and *G*_ST_ (genetic diversity among populations) in each locus.

Locus	Na	Ne	PIC	Ho	He	I	*F*_IT_ ^a^	*F*_IS_	*F*_ST_ ^a^	*D*_ST_	H_T_	*G*_ST_
**AHT4**	11.17	8.62	0.81	0.75	0.74	1.98	0.158	0.012	0.148	0.611	0.885	0.131
**AHT5**	11.50	9.28	0.78	0.88	0.84	2.16	0.040	−0.046	0.082	0.509	0.915	0.066
**ASB2**	11.33	9.20	0.77	0.91	0.85	2.18	−0.008	−0.073	0.061	0.363	0.904	0.044
**ASB17**	10.00	4.80	0.69	0.94	0.79	1.85	−0.146	−0.201	0.047	0.158	0.823	0.032
**ASB23**	11.83	6.77	0.74	0.96	0.85	2.13	−0.077	−0.132	0.049	0.260	0.890	0.033
**HMS1**	12.67	9.96	0.65	0.80	0.79	2.19	0.119	−0.016	0.132	0.657	0.910	0.116
**HMS2**	12.50	10.26	0.69	0.86	0.83	2.24	0.066	−0.033	0.096	0.575	0.918	0.079
**HMS3**	13.33	10.74	0.73	0.92	0.86	2.36	0.009	−0.059	0.065	0.452	0.923	0.048
**HMS6**	12.50	9.59	0.67	0.83	0.81	2.19	0.060	−0.022	0.119	0.661	0.920	0.103
**HMS7**	13.33	11.28	0.75	0.86	0.89	2.42	0.068	0.028	0.041	0.277	0.927	0.023
**HTG4**	12.67	9.80	0.71	0.89	0.85	2.26	0.022	−0.051	0.069	0.438	0.913	0.053
**HTG6**	12.83	9.86	0.68	0.77	0.82	2.23	0.151	0.058	0.099	0.559	0.912	0.081
**HTG7**	12.67	10.55	0.72	0.92	0.86	2.31	0.002	−0.064	0.062	0.423	0.919	0.046
**HTG10**	12.83	10.63	0.75	0.90	0.89	2.38	0.025	−0.015	0.040	0.277	0.927	0.022
**VHL20**	13.17	10.90	0.73	0.91	0.87	2.36	0.025	−0.032	0.055	0.385	0.924	0.037
**Mean** **(SE)**	12.29 (0.44)	9.48 (0.42)	0.73 (0.15)	0.87 (0.02)	0.84 (0.02)	2.22 (0.07)	0.037 (0.021)	−0.043 (0.016)	0.078 (0.009)	0.459 (0.459)	0.907 0.007	0.061 0.009

^a^ Wright’s statistics according to Weir and Cockerham [26].

**Table 3:** Hardy–Weinberg (HWS) equilibrium test for all studied microsatellite loci in the six paternal lineages.

Lineage	Locus														
	AHT4	AHT5	ASB2	ASB17	ASB23	HMS1	HMS2	HMS3	HMS6	HMS7	HTG4	HTG6	HTG7	HTG10	VHL20
**Zdravko**	0.803	0.892	0.785	0.046 *	0.075	0.991	0.214	0.977	0.909	0.695	0.509	0.062	0.370	0.222	0.386
**NONIUS XVII-30**	0.148	0.932	0.141	0.987	0.064	0.028 *	0.797	0.074	0.842	0.658	0.699	0.587	0.874	0.193	0.109
**Torpedo**	0.455	0.495	0.762	0.979	0.357	0.162	0.849	0.377	0.553	0.209	0.446	0.774	0.249	0.877	0.803
**Lider**	0.375	0.578	0.823	0.959	0.559	0.654	0.720	0.320	0.169	0.475	0.159	0.887	0.382	0.047 *	0.429
**Kalifa**	0.561	0.915	0.629	0.713	0.996	0.002 **	0.796	0.305	0.645	0.374	0.467	0.011 *	0.393	0.575	0.788
**Hrabar**	0.874	0.270	0.835	0.000 ***	0.006 **	0.370	0.292	0.121	0.921	0.325	0.471	0.052	0.275	0.837	0.792

Significant *p*-values (*p*  <  0.05 *, *p* <  0.01 **, *p* <  0.001 ***).

**Table 4:** Number of different alleles (Na), number of effective alleles (Ne), Shannon’s information index (I), observed (Ho) and expected (He) heterozygosity, number of private alleles unique to a single population (NPA), and Na freq. ≥ 5% (mean number of alleles for which the frequency is equal to or lower than 5%) in six paternal lineages of Danubian horse.

Lineage	Na	Ne	I	Ho	He	NPA	No. Different Alleles (Freq ≥ 5%)
**Zdravko**	4.20	2.73	1.07	0.65	0.57	-	3.06
**NONIUS XVII-30**	13.93	10.72	2.45	0.91	0.89	-	7.47
**Torpedo**	14.33	11.19	2.50	0.94	0.90	-	10.73
**Lider**	14.60	11.57	2.54	0.92	0.91	-	9.13
**Kalifa**	14.60	11.40	2.53	0.93	0.91	1	9.13
**Hrabar**	12.07	9.29	2.19	0.88	0.83	-	8.733
**Mean**	27.67	12.29	2.22	2.22	0.87	-	8.04

**Table 5:** Pairwise population matrix of Nei’s genetic distances (above the diagonal) and pairwise population *F*_ST_ values (below the diagonal) between six sire genealogical lineages.

	Zdravko	NONIUS XVII-30	Torpedo	Lider	Kalifa	Hrabar
**Zdravko**	0.000	1.142	1.055	0.960	1.023	0.660
**NONIUS XVII-30**	0.122	0.000	0.206	0.259	0.247	0.533
**Torpedo**	0.118	0.010	0.000	0.188	0.214	0.541
**Lider**	0.115	0.012	0.009	0.000	0.171	0.538
**Kalifa**	0.117	0.012	0.010	0.008	0.000	0.542
**Hrabar**	0.103	0.034	0.034	0.034	0.034	0.000

## Data Availability

Not applicable.

## References

[1] Gaidarska V.M., Ignatova M.M., Lytskanov P.I. (2017). Genetic resources of farm animals in Bulgaria—Conservation and management. Anim. Breed. Genet..

[2] Tanchev S. (2015). Conservation of genetic resources of autochthonous domestic livestock breeds in Bulgaria. A review. Bulg. J. Agric. Sci..

[3] Thornton P.K. (2010). Livestock production: Recent trends, future prospects. Philos. Trans. R. Soc. Lond. B Biol. Sci..

[4] Adelman M., Thompson K. (2017). Introduction to equestrian cultures in global and local contexts. Equestrian Cultures in Global and Local Contexts.

[5] Prescott W.H. (2018). Revival: History of the Conquest of Mexico: With a Preliminary View of the Ancient Mexican Civilisation and the Life of the Conqueror, Hernando Cortes.

[6] Klecel W., Martyniuk E. (2021). From the Eurasian steppes to the Roman circuses: A review of early development of horse breeding and management. Animals.

[7] Pinheiro M., Kjöllerström H.J., Oom M.M. (2013). Genetic diversity and demographic structure of the endangered Sorraia horse breed assessed through pedigree analysis. Livest. Sci..

[8] Librado P., Fages A., Gaunitz C., Leonardi M., Wagner S., Khan N., Hanghøj K., Alquraishi S.A., Alfarhan A.H., Al-Rasheid K.A. (2016). The evolutionary origin and genetic makeup of domestic horses. Genetics.

[9] Cressent M., Jez C. (2013). The French horse industry at present. Adv. Anim. BioSci..

[10] Leinonen R.M. (2016). From Servant to Therapist: The Changing Meanings of Horses in Finland.

[11] Marshall F.B., Dobney K., Denham T., Capriles J.M. (2014). Evaluating the roles of directed breeding and gene flow in animal domestication. Proc. Natl. Acad. Sci. USA.

[12] Druml T., Neuditschko M., Grilz-Seger G., Horna M., Ricard A., Mesarič M., Cotman M., Pausch H., Brem G. (2018). Population networks associated with runs of homozygosity reveal new insights into the breeding history of the Haflinger horse. J. Hered..

[13] Barzev G., Yordanov G., Yuseinov Y. (2005). The Bulgarian primitive horse in the area of Stara Planina mountain. Trakia J. Sci..

[14] Karaivanov R., Petrov A., Dobrev D., Tsankov T. (1971). Development, structure, exterior characteristics and standardization of the Danubian horse breed. Experian for Standardize of Horse Breeds in Bulgaria.

[15] Karaivanov R., Barzev G., Karadzhov T. (1989). Development and status of families in the Danubian horse breed. International Symposium on Half Breed Equine Breeding.

[16] ISAG/FAO Standing Committee Secondary Guidelines for Development of National Farm Animal Genetic Resources Management Plans. Measurement of Domestic Animal Diversity (MoDAD): Recommended Microsatellite Markers. http://dad.fao.org/cgi-bin/getblob.cgi?sid=ca53b91a6f7c80be8e7066f4a50.

[17] Binns M.M., Holmes N.G., Rolliman A., Scott A.M. (1995). The identification of polymorphic microsatellite loci in the horse and their use in thoroughbred parentage testing. Br. Vet. J..

[18] Bowling A.T., Eggleston-Stott M.L., Byrns G., Clark R.S., Dileanis S., Wictum E. (1997). Validation of microsatellite markers for routine horse parentage testing. Anim. Genet..

[19] Irvin Z., Giffard J., Brandon R., Breen M., Bell K. (1998). Equine dinucleotide repeat polymorphisms at loci ASB 21, 23, 25 and 37–43. Anim. Genet..

[20] Guérin G., Bertaud M., Amigues Y. (1994). Characterization of seven new horse microsatellites: HMS1, HMS2, HMS3, HMS5, HMS6, HMS7 and HMS8. Anim. Genet..

[21] Ellegren H., Johansson M., Sandberg K., Andersson L. (1992). Cloning of highly polymorphic microsatellites in the horse. Anim. Genet..

[22] Marklund S., Ellegren H., Eriksson S., Sandberg K., Andersson L. (1994). Parentage testing and linkage analysis in the horse using a set of highly polymorphic microsatellites. Anim. Genet..

[23] Haeringen H., Bowling A.T., Stott M.L., Lenstra J.A., Zwaagstra K.A. (1994). A highly polymorphic horse microsatellite locus: VHL20. Anim. Genet..

[24] Peakall R., Smouse P.E. (2012). GenAlEx 6.5: Genetic analysis in Excel. Population genetic software for teaching and research--an update. Bioinformatics.

[25] Nagy S., Poczai P., Cernák I., Gorji A.M., Hegedűs G., Taller J. (2012). PICcalc: An online program to calculate polymorphic information content for molecular genetic studies. Biochem. Genet..

[26] Weir B.S., Cockerham C.C. (1984). Estimating F-statistics for the analysis of population structure. Evolution.

[27] Yeh F.C., Yang R.-C., Boyle T.B.J., Ye Z.-H., Mao J.X. (1997). PopGene, the user-friendly shareware for population genetic analysis, molecular biology and biotechnology center. POPGENE User-Friendly Shareware Popul. Genet. Anal..

[28] Goudet J. (1995). FSTAT (Version 1.2): A computer program to calculate F-Statistics. J. Hered..

[29] Pritchard J.K., Stephens M., Donnelly P. (2000). Inference of population structure using multilocus genotype data. Genetics.

[30] Earl D.A., von Holdt B.M. (2012). STRUCTURE HARVESTER: A website and program for visualizing STRUCTURE output and implementing the Evanno method. Conserv. Genet. Resour..

[31] Evanno G., Regnaut S., Goudet J. (2005). Detecting the number of clusters of individuals using the software STRUCTURE: A simulation study. Mol. Ecol..

[32] Kopelman N.M., Mayzel J., Jakobsson M., Rosenberg N.A., Mayrose I. (2015). Clumpak: A program for identifying clustering modes and packaging population structure inferences across K. Mol. Ecol. Resour..

[33] Rosenberg N.A. (2004). DISTRUCT: A program for the graphical display of population structure. Mol. Ecol. Notes.

[34] Karaivanov R. (1971). Development, structure, exterior features and standardization of the Danube breed. An attempt to standardize horses in Bulgaria. Zhivotnovudstvo.

[35] Karaivanov R. (1975). Origin, Genealogical Structure and Development of the Danube Horse Breed. Ph.D. Thesis.

[36] Moravcikova N., Kasarda R., Kukuckova V., Vostry L., Kadlecík O. (2016). Genetic diversity of Old Kladruber and Nonius horse populations through microsatellite variation analysis. Acta. Agric. Slov..

[37] Lukanova N., Vlaeva R., Hristova D., Georgieva S., Barzev G. (2015). Study on the genetic diversity of Trotter horses populations in Bulgaria. Agricul. Sci..

[38] Barzev G., Zhelyazkov E., Barzeva V., Hristova D., Sabev Z. (2010). Genetic diversity in Bulgarian Thoroughbred using microsatellite DNA markers. Agric. Sci. Technol..

[39] Zeng L., Chen N., Yao Y., Dang R., Chen H., Lei C. (2019). Analysis of genetic diversity and structure of Guanzhong horse using microsatellite markers. Anim. Biotechnol..

[40] Cosenza M., La Rosa V., Rosati R., Chiofalo V. (2019). Genetic diversity of the Italian Thoroughbred horse population. Ital. J. Anim. Sci..

[41] Fornal A., Kowalska K., Zabek T., Piestrzynska-Kajtoch A., Musiał A.D., Ropka-Molik K. (2020). Genetic diversity and population structure of Polish Konik horse based on individuals from all the male founder lines and microsatellite markers. Animals.

[42] Seyedabadi H.R., Sofla S.S. (2017). Microsatellite analysis for parentage verification and genetic characterization of the Turkmen horse population. Kafkas Univ. Vet. Fak. Derg..

[43] Shannon C.E. (1948). A mathematical theory of communication. Bell Syst. Tech. J..

[44] Vostry L., Vostra Vydrova H., Hofmanova B., Vesela Z., Majzlik I. (2015). Genetic diversity in Czech Haflinger horses. Poljoprivreda.

[45] Grilz-Seger G., Druml T., Neuditschko M., Dobretsberger M., Horna M., Brem G. (2019). High-resolution population structure and runs of homozygosity reveal the genetic architecture of complex traits in the Lipizzan horse. BMC Genom..

[46] Funk S.M., Guedaoura S., Juras R., Raziq A., Landolsi F., Luís C., Martínez A.M., Musa Mayaki A., Mujica F., Oom M. (2020). do M.; et al. Major inconsistencies of inferred population genetic structure estimated in a large set of domestic horse breeds using microsatellites. Ecol. Evol..

[47] Fornal A., Kowalska K., Zabek T., Piestrzynska-Kajtoch A., Musiał A.D., Ropka-Molik K. (2021). Genetic variability and population structure of Polish Konik horse maternal lines based on microsatellite markers. Genes.

[48] Popova M., Lukanova N., Vlaeva R. (2020). Monitoring of the sire lines of the Danube horse breed. Anim. Sci..

[49] Karadzhov T. (1997). Influence of Some Genetic and Non-Genetic Factors on Reproductive Performance and Exterior Measurements in Pleven and Danube Horses. Ph.D. Thesis.

